# Faces do not guide attention in an object-based facilitation manner

**DOI:** 10.3758/s13414-023-02742-0

**Published:** 2023-06-22

**Authors:** Tong Xie, Shimin Fu, Giovanni Mento

**Affiliations:** 1 Department of General Psychology, University of Padova, Via Venezia, 8, 35131 Padova, Italy; 2 Department of Psychology and Center for Brain and Cognitive Sciences, School of Education, Guangzhou University, Guangzhou, 510006 China; 3 IRCCS E. Medea Scientific Institute, Treviso, Italy

**Keywords:** Face, Object-based attention, Double-rectangle paradigm, Filtering cost

## Abstract

Numerous studies on face processing have revealed their special ability to affect attention, but relatively little research has been done on how faces guide spatial attention allocation. To enrich this field, this study resorted to the object-based attention (OBA) effect in a modified double-rectangle paradigm where the rectangles were replaced with human faces and mosaic patterns (non-face objects). Experiment 1 replicated the typical OBA effect in the non-face objects, but this effect was absent in Asian and Caucasian faces. Experiment 2 removed the eye region from Asian faces, but still found no object-based facilitation in the faces without eyes. In Experiment 3, the OBA effect was also observed for faces when the faces disappear a short period before the responses. Overall, these results revealed that when two faces are presented together, they do not exert object-based facilitation regardless of their facial features such as race and the presence of eyes. We argue that the lack of a typical OBA effect is due to the filtering cost induced by the entire face content. This cost slows down the response when attention shifts within a face and results in the absence of object-based facilitation.

## Introduction

Faces are the most common and vital stimuli perceived by humans as they carry a multitude of information essential for life (Oruc, Balas, & Landy, 2019). Much research on face recognition (Hancock & Rhodes, 2008), detection (Van Rullen, 2006), or categorization (Zhao & Bentin, 2008) has targeted specific effects such as those elicited by face inversion (McKone & Yovel, 2009), other-race (Young et al., 2012), and face holistic processing (Richler & Gauthier, 2014). These studies provided extensive knowledge of the mechanisms underlying face processing. In contrast, there has been little research on how faces guide spatial attention compared to non-face objects.

It is well accepted that attention can be guided by objects, a phenomenon known as *object-based attention* (OBA). The OBA has been mostly studied through the well-established double-rectangle paradigm (Egly et al., 1994). In this paradigm, two rectangles are oriented either vertically or horizontally with their vertices at the endpoints of a virtual square. A spatial cueing task is deployed where the cue and target can appear at the corners of these two rectangles, but they never appear diagonally. Three critical conditions are determined by the relationship between the cue and target: in the *valid* condition, the target appears at the cued location; in the *within* and *between* conditions the target appears at another corner of the cued rectangle or the near end of the other one, respectively. Typical results show the spatial validity effect, characterized by shorter response times (RTs) in the valid than invalid conditions. Moreover, they also reveal faster responses in the *within* condition than the *between* condition. This difference in RT is referred to as the OBA effect.

Follow-up studies have replaced rectangles with various objects and have found that the OBA effect is not limited to objects formed by geometric shapes. Instead, it can be widely observed in different kinds of objects. For example, the OBA effect has been demonstrated with Gestalt-law-based objects (Marino & Scholl, 2005; Marrara & Moore, 2003), memory or imaginative objects (Ongchoco & Scholl, 2019; Xie et al., 2021), objects encoded with social or semantic information (Li & Logan, 2008; Yin et al., 2018), and objects in real-world scenes (Malcolm & Shomstein, 2015). This wealth of evidence suggests that attention can be guided by various objects. While the OBA effect has undoubtedly been demonstrated in these non-face objects, there is as yet no sufficient evidence on whether faces affect attention in an object-based manner.

Neuroimaging studies have revealed that the face can be selected by attention as a processing unit as non-face objects do (Baldauf & Desimone, 2014; Cohen & Tong, 2015; O'Craven et al., 1999; Serences et al., 2004). For example, O'Craven et al. (1999) presented an overlapping house and face and asked participants to pay attention to either the face or the house. During this task, one of the objects oscillated while the other remained static. The results revealed that the motion-related brain region was only activated when the attended object oscillated, indicating that the activation of the motion-related brain region was contingent upon attention being directed to the oscillating face or house.

In behavior experiments, evidence from the composite-face paradigm (Young et al., 1987) has also proved that faces can be processed in an object-based manner (Curby et al., 2016). This paradigm presents two composite faces in succession and asks participants to determine whether the top halves of the two faces are the same or different. The first composite face consists of two halves of the same face, but the second composite face can consist of two halves from either the same face or different faces. The results showed a composite effect that participants succeeded better (manifested by a higher sensitivity *d’* or shorter RTs) at detecting the change in the second face when it consisted of the same face, compared to when it consisted of different halves. However, when the second composite face was misaligned by laterally offsetting its bottom half, the composite effect was reduced because the misaligned faces broke the integrity of the face (Curby & Entenman, 2016; Zhao et al., 2016). Therefore, the composite effect supported the hypothesis that faces are processed holistically (Young et al., 2012). Curby et al. (2016) proposed that the holistic processing of faces aligns with the concept of OBA, where the cohesiveness or "objecthood" of an object is crucial for OBA mechanisms (MaTSukura & Vecera, 2006). Furthermore, these results in the composite-face paradigm are consistent with the attentional-spreading hypothesis (Chen & Cave, 2008; Richard et al., 2008), which suggests that attention spreads within an object and facilitates attentional operations within that object. In the aligned composite face condition, attention automatically spreads throughout the entire face, leading to interference between task-relevant and task-irrelevant halves of the face (Curby et al., 2016). However, although the evidence in the overlapping stimuli and composite-face paradigms suggests that attention can select a face as an attentional unit, it is not yet clear how the faces guide spatial attention compared to non-face objects.

Only in recent years have researchers started using faces in the double-rectangle paradigm, which specifically aims to investigate the mechanisms underlying spatial attention allocation. In these studies, the two rectangles were replaced by two human faces and the OBA effect was measured by the difference between the *within* and *between* conditions. However, these studies encountered challenges in reliably demonstrating the OBA effect with human faces (e.g., Valenza & Calignano, 2021; Valenza et al., 2014; Xie et al., 2022; but see Hu et al., 2021; Song et al., 2021; Yan et al., 2022).

Hu et al. (2021) and Song et al. (2021) found significant OBA effects in faces when focusing on the role of facial expressions and eye gaze. They reached the conclusion that facial expressions and eye gaze can modulate the magnitude of the OBA effect. These conclusions imply an indication that faces can elicit the OBA effect. Since the OBA effect has been widely replicated in real-world objects (e.g., Malcolm & Shomstein, 2015; Zhao et al., 2020), as a kind of real-world object featuring general object properties, faces are justified in eliciting the OBA effect (Hu et al., 2021; Song et al., 2021). From a more basic perspective, Yan et al. (2022) suggested that faces containing direct eye gaze would capture attention and result in faster RTs in the *within* condition, which leads to the OBA effect in faces (Yan et al., 2022).

However, our previous works found no OBA effect when human faces were used to fill the rectangles. The results revealed longer RTs when shifting attention within a face than in a non-face object, which suggested that the null OBA effect in faces was caused by the slower shifting attention within a face (Xie et al., 2022). Likewise, Valenza et al. (2014, 2021) found a null OBA effect in faces. The authors offered an interpretation for these results. Faces are highly biologically and socially significant visual stimuli in the human environment (Palermo & Rhodes, 2007), and it could be expected that they receive enhanced processing, enabling rapid shifting of attention between faces. When multiple faces are present, the viewer's focus of attention may encompass all the faces, leading to shorter saccade latencies in the between condition and thus diminishing the OBA effect. However, it should be considered that Valenza et al. (2014) measured saccade latencies instead of RTs. This methodological difference may have introduced a measurement bias, and induced different interpretations of the underlying mechanism. In summary, the question of whether human faces can elicit the OBA effect in the double-rectangle paradigm remains controversial, and further experimental validation is needed to explore potential explanations.

Furthermore, it is worth mentioning the influence of rectangle orientation on the OBA effect. Although in the seminal double-rectangle paradigm Egly et al. (1994) found that the rectangle orientation did not interact with the OBA effect, several later studies suggested that the typical OBA effect is only found in the horizontally oriented rectangles, but that it turns to be null (no significant differences when comparing RTs in the *within* and *between* conditions) or reversed (faster RTs in the *between* than in the *within* conditions) in the vertically oriented rectangles (Chen & Cave, 2019; Greenberg et al., 2014; Pilz et al., 2012). These phenomena can be attributed to the orientation bias positing that attention is easier to engage horizontally than vertically (Thornton et al., 2021). Specifically, if the rectangles are oriented vertically, the *within* always corresponds to the vertical engagement of attention, and the *between* always corresponds to the horizontal engagement of attention. The opposite occurs for horizontally oriented rectangles. For this reason, the horizontal bias of attention would undermine the OBA effect in vertically oriented rectangles but exacerbate it when they are horizontally displaced. To rule out the influence of horizontal bias, OBA studies using the double-rectangle paradigm generally counterbalance the rectangle orientations. However, using rotated faces to partial out the orientation bias may have undesired implications, such as disrupting the natural physical features of this stimulus. To overcome any potential loss of ecological validity due to presenting faces in an unnatural, horizontal orientation, our previous study proposed a method to partial out the horizontal bias of attention in the double-rectangle paradigms if only deploying vertically oriented rectangles (Xie et al., 2022). This method is well suited to rule out any potential horizontal bias from behavioral experiments. Specifically, this method is based on first obtaining RTs in the baseline condition where no object is presented. Then, the horizontal bias is obtained at the individual level by subtracting the RT in the horizontal attentional shift from the vertical attentional shift. This bias allows us to calculate a *corrected* OBA effect, which differs from the traditionally calculated one as it takes into account the weight of stimuli orientation on attention shifting. Remarkably, even after ruling out any confounding effect of the horizontal bias, our previous study did not find the OBA effect in faces (Xie et al., 2022).

Considering the particularity of faces, two essential factors may account for this null finding. The first factor may depend on the specific affective and cognitive relevance of faces. It is noteworthy that our previous study presented only Asian faces to Asian participants. However, same-race faces are of more subjective importance and lead to a more effortful encoding while other-race faces induce more superficial processing (Lingyun et al., 2007; Meissner et al., 2005). By contrast, other-race faces may be more likely to elicit the OBA effect because they may tend to be processed more like non-face objects. Note that the aforementioned studies failed to find the OBA effect in faces using the same-race faces as material. Thus, it is interesting to ask whether the OBA effect can survive in the other-race faces. The second factor may be the critical role of the eye region in face processing, as it is the most attended area within the face (Farroni et al., 2002; Itier et al., 2007; Itier & Batty, 2009). It is therefore reasonable to assume that the removal of the eyes may deprive a face of its features, and hence the OBA effect would be found in faces without eyes.

To verify the two hypotheses outlined above, we conducted two experiments. In Experiment 1, we tested whether the race of faces could modulate the OBA effect by presenting Chinese participants with same-race (Asian) and other-race (Caucasian) faces. In Experiment 2, we examined the role of eyes by removing the eye region from intact Asian faces. Based on our previous work, which demonstrated a null OBA effect with same-race faces (Xie et al., 2022), and the knowledge that eyes are essential for facial processing (Itier et al., 2007), we hypothesized that we could replicate the null OBA effect in Asian faces and observe the presence of the OBA effect in Caucasian faces (H1), and would find a significant OBA effect in the faces without eyes (H2).

Contrary to our assumptions, neither Experiment 1 nor Experiment 2 provided evidence of the OBA effect, disconfirming any causal role of race (H1) or eye presence (H2) for previous null results. In light of this, we decided to investigate the possibility of null results being influenced by face-dependent attentional filtering cost (Chen, 2000; Chen et al., 2020; Folk & Remington, 1998; Kahneman et al., 1983). The filtering cost account suggests that the presence of task-irrelevant items could slow the deployment of attention to the targets by requiring an effortful and time-consuming filtering operation (Theeuwes, 2010). Experimentally, this cost results in longer RTs (Folk & Remington, 1998). In the context of our study, faces contain more complex information compared to non-face objects, which may incur a higher filtering cost and subsequently lead to longer RTs when shifting attention within a face. Consequently, the null (or even reversed) OBA effect in faces could be attributed to the filtering out of the entire face content. To minimize this potential influence, in the third experiment (Experiment 3), we manipulated the trial structure to avoid any spatial and temporal overlapping between task-irrelevant (faces) and task-relevant (target letters) items. This manipulation was achieved by simply shortening the face permanence on the screen so that it disappeared before the appearance of the target stimulus. In this way, we ensured eliminating (or at least dramatically reducing) any stimulus-specific filtering cost on attentional shifting. Furthermore, based on a finding that objects can still elicit the OBA effect after their short period of offset (Xie et al., 2021), we predict that the OBA effect would be observed for faces that disappear for a short period (H3).

## Experiment 1

Experiment 1 was designed to test whether the race of faces could modulate the OBA effect in the context of a modified double-rectangle paradigm. We recruited Asian participants from China (see below for details) and filled the rectangles with mosaics, Asian or Caucasian faces. Mosaic objects, representing non-face objects, are used as control conditions that are supposed to elicit the OBA effect. Based on H1, we expect the OBA effect to be absent in Asian faces but present in Caucasian faces, or, alternatively, to find a significant interaction between race and the OBA effect.

In addition, given the particularity of the face, the rectangles were presented only vertically, and both the traditional and the corrected (i.e., RTs after excluding the horizontal bias of attention as described above) OBA effect were considered. Although the faces were only vertically oriented, they can be upright or inverted in this study. Given that the inverted faces basically disrupt the holistic property of a face (Tanaka & Simonyi, 2016), this design also allows us to test whether the holistic process of the face can affect the OBA effect.

### Method

#### Participants

A priori power analysis by G*Power 3.1 (Faul et al., 2007) determined that a sample size of 36 showed a middle effect size (0.25) and a test force (1-β) of 0.9 in a 2 × 3 within-subject repeated-measures ANOVA. However, in line with our previous online study (Xie et al., 2022), this study adopted a larger sample size (at least 70 participants, same scale as the previous experiments) to override any potential effect underestimation due to online data collection (Del Popolo Cristaldi et al., 2022).

Seventy-nine participants (aged 20.3 ± 1.7 years, 16 males, two left-handed) from Guangzhou University were recruited for Experiment 1, with a reward of 7 Yuan or course credit. All participants had normal or corrected-to-normal vision (self-reported) and were naïve to this experiment. Each participant voluntarily enrolled and read an informed consent form before the experiment. This project was approved by the Ethics Review Committee (Institutional Review Board) of the Education School, Guangzhou University, and was conducted according to the principle of ethics (Protocol code GZHU2020010).

#### Stimuli and materials

**Fig. 1:**
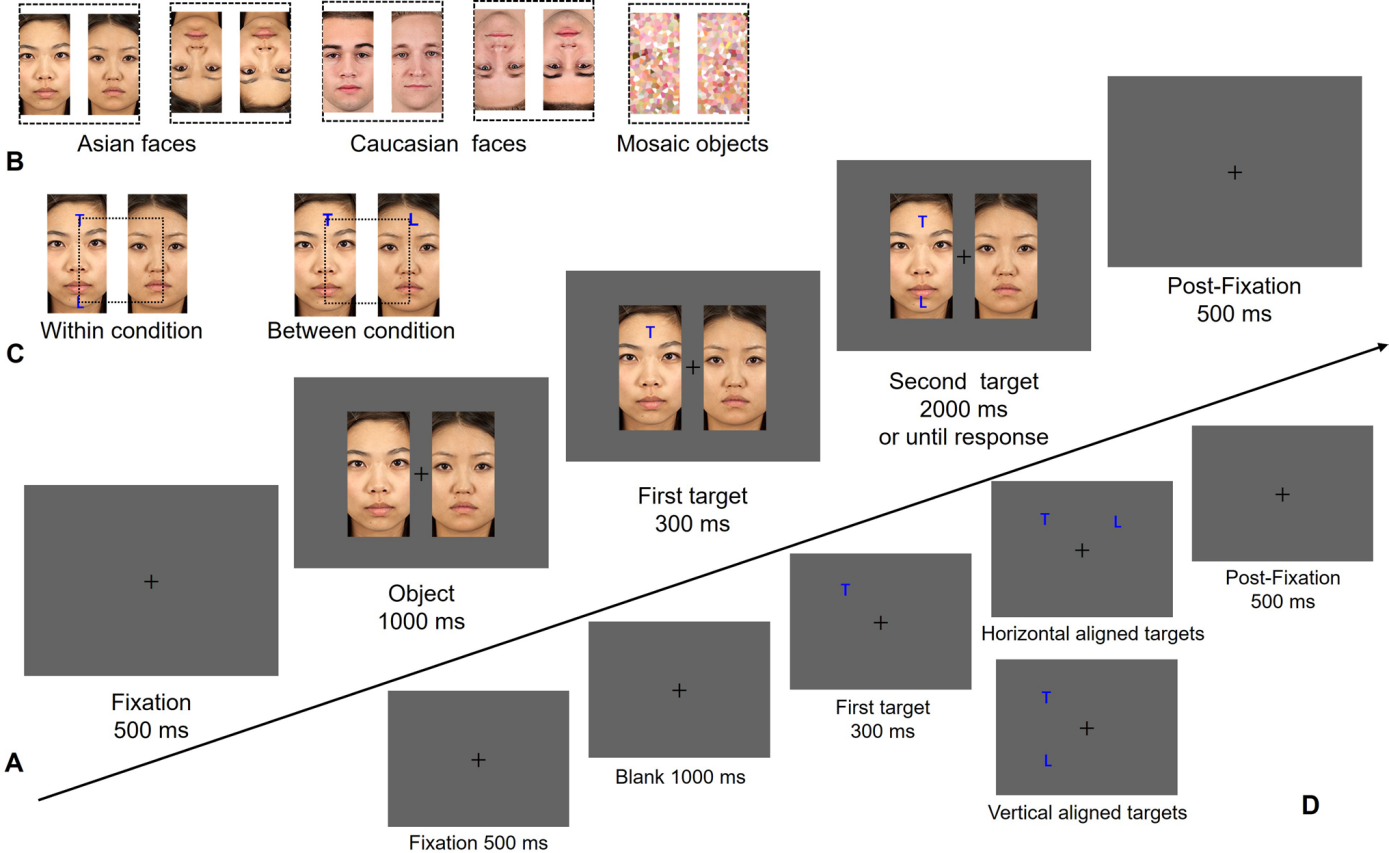
Panel **A** illustrates the procedure of each trial. Panel **B** displays the configuration of Asian faces, Caucasian faces, and mosaic objects. Panel **C** displays the *within* and *between* conditions. Panel **D** illustrates the baseline correction procedure to rule out the horizontal bias of attentional shifting. The dashed square is for presenting the stimuli location and did not show in the experiment. The faces are reproduced with permission from the Chicago Face Database (Ma et al., 2015)

The parameters reported in this study were based on a screen size of 33 × 18.5 cm, with a viewing distance of 60 cm. The stimuli and trial sequence are shown in Fig. 1. The fixation was black and subtended 0.5°. The rectangle subtended 8.3° × 16.5°, with different content depending on the condition. The targets were blue (RGB: 0, 0, 255) uppercase letters T and L and subtended 2°. The error feedback was a red cross subtending 4° × 4°. All stimuli were displayed on a gray background (RGB: 100, 100, 100).

The face materials were selected from the Chicago Face Database (Ma et al., 2015). Experiment 1 included 32 Caucasian faces and 32 Asian faces, half female and half male. The faces were cropped to rectangles with an aspect ratio of 2:1. Two different faces of the same gender and race formed a combination with upright or inverted orientation. The mosaic objects were obtained by crystallizing the face images in Photoshop.

#### Apparatus

Stimuli presentation and manual response measurements were controlled by Open Sesame (Mathôt et al., 2012). The experiment was carried out online through the JATOS hosting server (Lange et al., 2015).

#### Design and procedure

Experiment 1 was a 2 (objecthood: *between* vs. *within*) × 3 (object type: Asian, Caucasian, mosaic) within-subject design. The objecthood was determined by two targets: the *within* condition consisted of two targets appearing within the same object, while the *between* condition was when two targets appeared in different objects. The object type was the content of the objects (Caucasian face, Asian face, or mosaic). The mosaic objects were included as the control condition for comparing the OBA effect between faces and non-face objects. In addition, this experiment contained a baseline condition (shown in Fig. 1D) in which no objects were presented, which aimed at testing the horizontal bias. That is, in the baseline condition, two targets appeared in the background with the same time parameters as other conditions. All conditions were intermixed in the experiment, which consisted of a total of 256 trials (32 trials of *within* in Asian faces, 32 trials of *between* in Asian faces, 32 trials of *within* in Caucasian faces, 32 trials of *between* in Caucasian faces, 32 trials of *within* in mosaic objects, 32 trials of *between* in mosaic objects, and 64 trials of baseline condition). Two correct responses (same vs. different) and four locations of the first target (up-left, down-left, up-right, down-right) were also balanced and randomly intermixed in the whole experiment.

Instead of using the detection or discrimination task, our study resorts to the two sequential targets comparison task (Lamy & Egeth, 2002), which was also adopted in previous studies and proved to successfully elicit the OBA effect (Xie et al., 2022). The advantage of this task is that the appearance of two targets eliminates the valid trial included in the original detection version and also avoids the influence of attentional strategy brought by cue validity (Shomstein & Behrmann, 2008). Thus, this task allows us to save a considerable number of trials to shorten the duration of online data collection.

Each trial began with a 500-ms fixation followed by a 1,000-ms object presentation. Then, two targets appeared sequentially with a stimulus onset asynchrony (SOA) of 300 ms. The two targets and objects were maintained on the screen until response or for a maximum of 2,000 ms. The trial ended with another 500-ms fixation.

The task was to judge whether the two targets were the same or not, by pressing the keyboard “F” or “J,” which were counterbalanced between participants. Participants were required to maintain their eyes on the fixation during the experiment and to respond both quickly and correctly. Before going through the formal test, all participants were required to pass a practice session. The task in the practice session was the same as the formal test and would end after eight correct responses in a row. In the formal test, the 256 trials were divided into four blocks, with self-terminal breaks. The whole experiment lasted about 13 min.

Before starting an online experiment, participants received a PDF file with instructions and requirements for informed consent. The instruction required them to (1) close all irrelevant software and maintain a solid internet connection; (2) set the screen resolution at 1,024 × 768 pixels or the same ratio; (3) keep their eyes in front of the screen at about 60 cm; and (4) run the experiment in a quiet and undisturbed environment. After the participants read this file, we provided them with the experimental URL and assigned each participant a subject ID. The forced full screen was turned on throughout the experimental session.

### Results and analyses

Participants whose experimental duration exceeded 25 min (one participant) and accuracy was lower than 85% (four participants) were excluded. Therefore, only 74 participants were included in the analyses.

Overall, incorrect responses (5.0%), RTs faster than 150 ms or slower than 1,000 ms (4.6%), and RTs outside 2 standard deviations (SDs) of each condition (4.2%) were discarded. Thus, 86.2% of the total trials were included in the statistical model. For all the experiments in this study, the Greenhouse–Geisser correction was applied when the assumption of sphericity was violated. In this case, the corrected *p*-value was reported. The *p*-values in post hoc comparisons were Holm-corrected.

### Traditional object-based attention (OBA) analysis

**Fig. 2:**
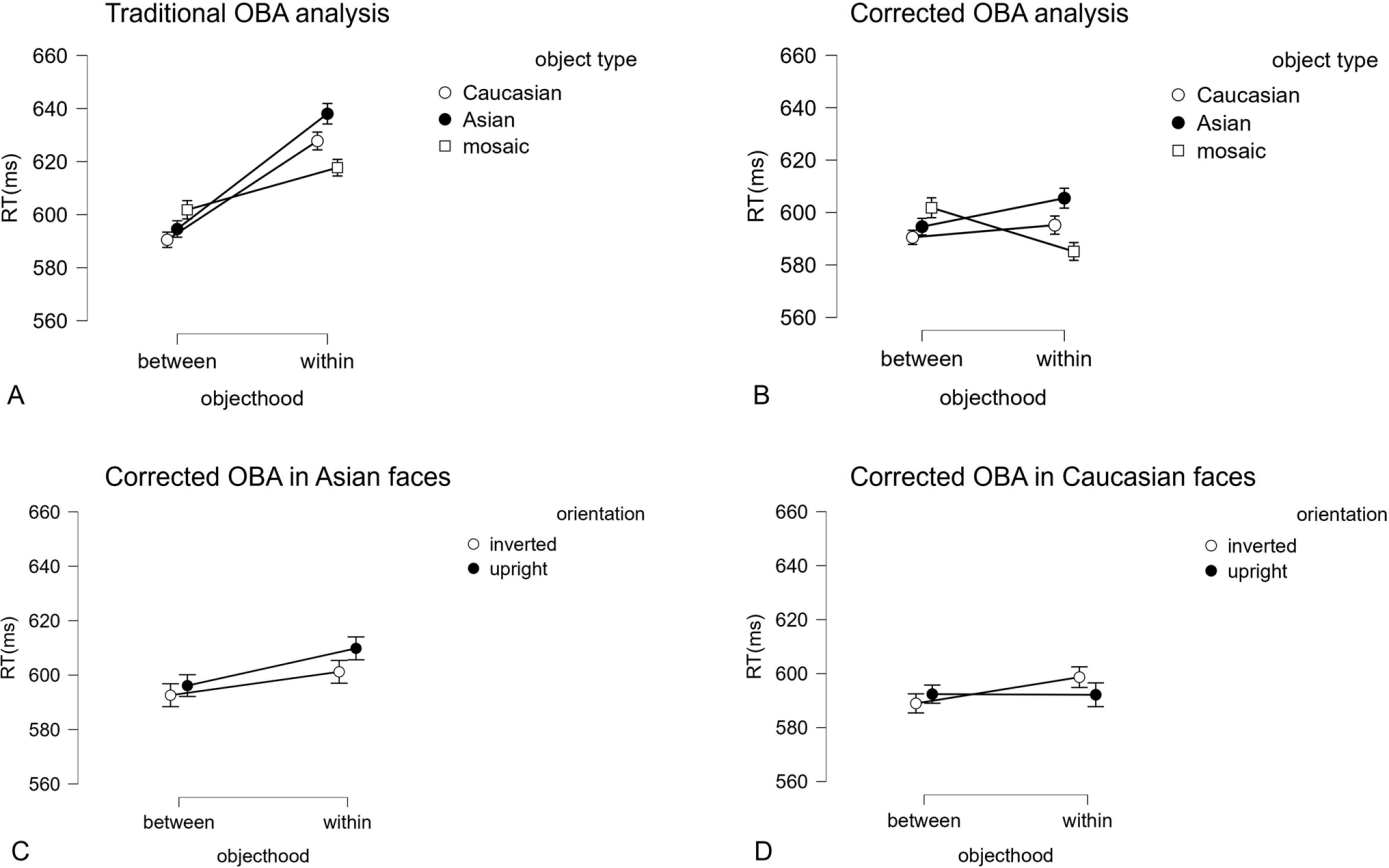
The line chart displays the results of Experiment 1. Panel **A** shows the traditional OBA analysis. Panel **B** shows the corrected OBA analysis. Panels **C** and **D** are the corrected OBA analysis in Asian and Caucasian faces, with facial orientation as a factor

A 2 × 3 repeated-measures ANOVA was conducted on RTs, with objecthood (*within* vs. *between*) and object type (Asian vs. Caucasian vs. mosaic) as within-subject factors. The results are shown in Fig. 2 (A). The main effect of objecthood was significant, *F*(1,73) = 67.08, *p* < .001,* η*_*p*_^2^ = .479, with the shorter RTs in the *between* (596 ± 8 ms) than in the *within* (628 ± 10 ms) condition, showing the reversed OBA effect. The main effect of object type was significant, *F*(2, 146) = 4.31, *p* = .019, *η*_*p*_^2^ = .056. The post hoc test revealed that RTs in Asian faces (616 ± 9 ms) were longer than Caucasian faces (609 ± 9 ms), *t*(73) = 3.19, *p* = .006, Cohen’s *d* = .37. The interaction was significant, *F*(2, 146) = 12.34, *p* < .001, *η*_*p*_^2^ = .145, suggesting the OBA effect is modulated by the object type. As expected, the traditional analysis showed the reversed OBA effect, suggesting the influence of horizontal bias. Thus, we resort to the corrected OBA analysis.

### Horizontal bias

The horizontal bias was calculated in the baseline condition, where attention had to be engaged either horizontally or vertically depending on whether the two targets are aligned horizontally or vertically. A one-way repeated-measures ANOVA was conducted on the RTs, with the orientation of attentional engagement (horizontal vs. vertical) as within-subject factors. The results showed that RTs were shorter when attention was engaged horizontally (609 ± 8 ms) than vertically (641 ± 10 ms), *F*(1, 73) = 57.67, *p* < .001, *η*_*p*_^2^ = 0.441, showing a mean horizontal bias of 32 ms.

### Corrected OBA analyses

To partial out the individual horizontal bias from the OBA effect, the *corrected within* condition was considered, which was defined as the RT of each participant in the *within* condition minus their own horizontal bias. Consequently, the corrected OBA effect was RT in the *between* subtracts RT in the *corrected within* condition.

A new 2 × 3 repeated-measures ANOVA was then conducted on RTs, with objecthood (*corrected within* vs. *between*) and object type (Asian vs. Caucasian vs. mosaic) as within-subject factors. The results are shown in Fig. 2 (B). This analysis confirmed that the main effect of objecthood was no longer significant, *F*(1,73) = 0.008, *p* = .928, *η*_*p*_^2^ < .001, indicating the absence of the corrected OBA main effect. The main effect of object type, *F*(2, 146) = 4.31, *p* = .019, *η*_*p*_^2^ = .056, as well as the interaction was still significant, *F*(2, 146) = 12.34, *p* < .001, *η*_*p*_^2^ = .145. The post hoc tests were conducted on the object type in Asian, Caucasian, and mosaic. For Asian faces, RTs were not different in *between* (595 ± 8 ms) and *corrected within* (605 ± 10 ms), *t*(73) = –2.02, *p* = .404, indicating the absence of corrected OBA effect. Likewise, for Caucasian faces, RTs were not different in *between* (591 ± 8 ms) and *corrected within* (595 ± 9 ms) conditions, *t*(73) = –0.81, *p* = 1, indicating again the absence of corrected OBA effect. Yet, for mosaic objects, RTs were shorter in *corrected within* (585 ± 8 ms) than *between* (602 ± 9 ms) conditions, *t*(73) = –3.10, *p* = .032, demonstrating that the OBA effect survived the correction when tested with non-face objects. Another 2 × 2 repeated-measures ANOVA was conducted to test whether the race (Asian vs. Caucasian) modulate the corrected OBA effect. The interaction was not significant, *F*(1, 73) = 1.12, *p* = .29, *η*_*p*_^2^ = .015, indicating that the corrected OBA effect was not affected by race.

### Facial orientation

To further test whether face orientation has different influences on the corrected OBA effect, a 2 × 2 × 2 repeated-measures ANOVA was conducted on RTs, with objecthood (*corrected within* vs. *between*), object type (Asian vs. Caucasian), and face orientation (upright vs. inverted) as within-subject factors. The results are shown in Fig. 2 (C and D). The analysis showed that the main effect of the orientation and its interactions with race and objecthood were not significant (the statistical data are available at online material), suggesting that after excluding the horizontal bias, race did not influence the OBA even when considering their upright vs. inverted orientation as a potential source of attentional bias.

#### ACC analyses

Although this study focused on RT data, the ACC analyses were also conducted in all experiments, which showed no indication of the speed-accuracy trade-off.

A 2 × 3 repeated-measures ANOVA was conducted on the ACC, with the objecthood (*within* vs. *between*) and object type (Asian, Caucasian, and mosaic) as within-subject factors. The main effect of the objecthood was significant, *F*(1, 74) = 14.36, *p* < .001, *η*_p_^2^ = .164, with a higher ACC in *between* (95.6 ± 0.5%) than *within* (94.0 ± 0.6%) conditions, indicating the reversed OBA effect. The main effect of object type and interaction were not significant.

In the baseline condition, a one-way repeated-measures ANOVA was conducted on the ACC, with the orientation of attentional engagement (horizontal vs. vertical) as within-subject factors. The main effect was significant, with a higher ACC in the horizontal engagement (96.6 ± 0.5%) than in vertical engagement (95.0 ± 0.5%), *F*(1,74) = 7.34, *p* = .008, *η*_p_^2^ = .091, confirming the presence of a horizontal bias. The corrected OBA was analyzed similarly to RT data (*corrected within* was the *within* plus horizontal bias). No significant main effect or interaction was found.

In short, for the ACC data, the traditional OBA analysis showed the reversed OBA main effect but the corrected OBA analysis found neither OBA main effect nor interaction.

## Discussion

In a modified double-rectangle paradigm, through the traditional OBA analysis, we found reversed OBA effects in Asian faces, Caucasian faces, and even in non-face objects (mosaic patterns). After ruling out the confounding role of horizontal bias (Xie et al., 2022), the (corrected) OBA effect was observed in the mosaic objects, as expected, but it was still absent in both Asian and Caucasian faces. These results indicated that faces, regardless of their race, could not elicit the OBA effect. In addition, the upright and inverted faces did not affect the OBA effect, suggesting that the holistic property of faces can not explain the null OBA effect in faces.

## Experiment 2

In Experiment 1, we found that faces did not elicit the OBA effect for either Asian or Caucasian faces. However, it left unaddressed the question of why the OBA effects were absent. Since the results of Experiment 1 suggested that the holistic property of faces may not be the reason why the OBA effect is absent, Experiment 2 extended upon these findings by focusing on the role of specific facial features.

In this regard, numerous studies have suggested that the eyes are the most critical feature of face processing (Itier et al., 2007; Royer et al., 2018). According to this view, a face without the eye region may be perceived as more like a non-face object, and as such should be more likely to elicit the OBA effect. This experiment aimed at addressing this hypothesis by introducing eyeless faces to investigate whether the eyes are responsible for the absent OBA effect. As aforementioned, we hypothesized (H2) that if the eyes were a core face feature contributing to the absent OBA effect, this effect should be observed in the eyeless faces.

### Method

#### Participants

Seventy-five participants (aged 20.2 ± 1.5 years, 15 males, six left-handed) from Guangzhou University were recruited for this experiment, with a reward of 5 Yuan or course credit. All participants have normal or corrected-to-normal vision (self-reported) and were naïve to this experiment. Each participant voluntarily enrolled and read an informed consent form before the experiments.

#### Design and procedure

**Fig. 3:**
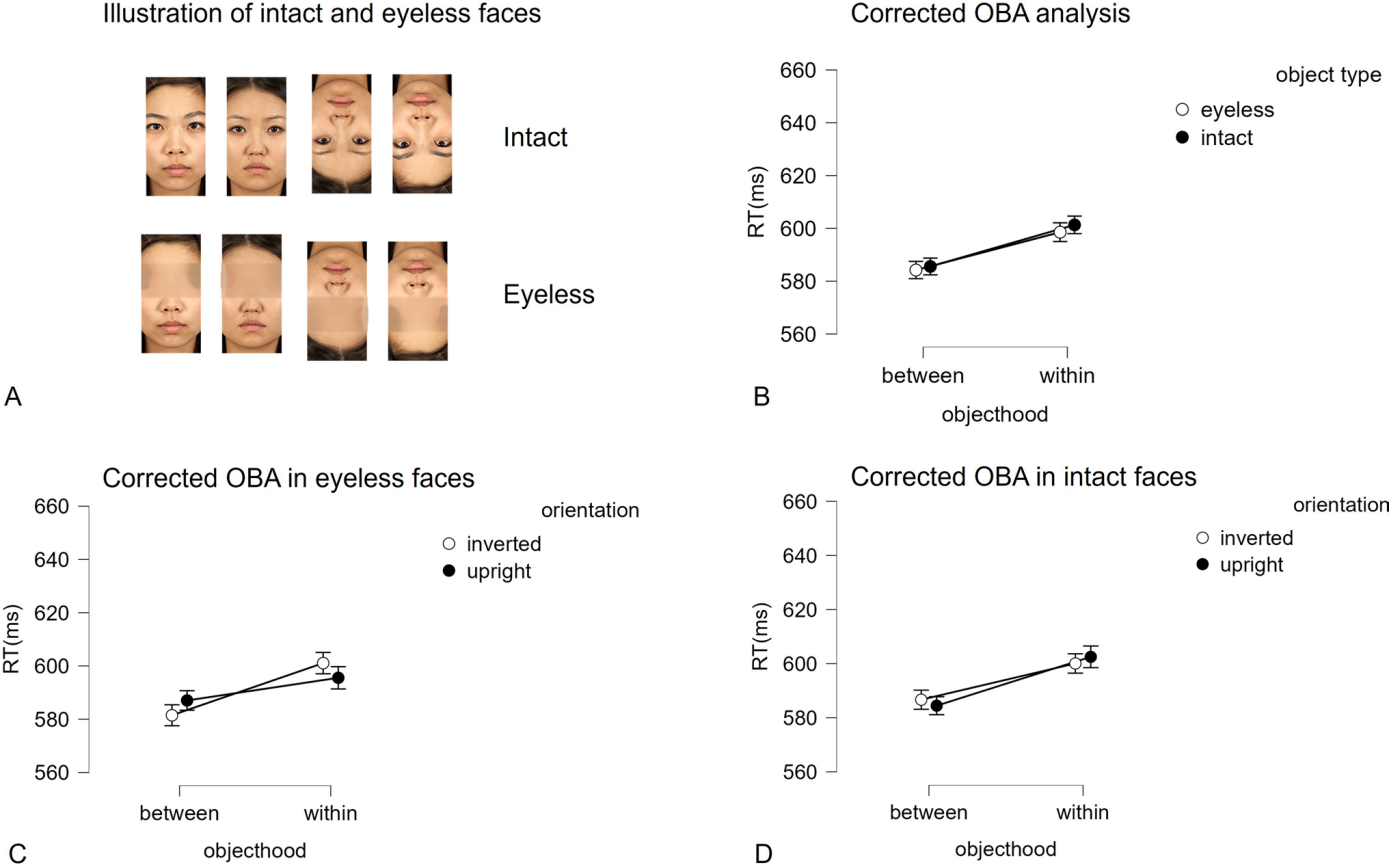
Panel **A** illustrates the intact and eyeless conditions. Panel **B** displays the corrected object-based attention (OBA) analysis. Panels **C** and **D** are the corrected OBA analysis in eyeless and intact conditions, with facial orientation as a factor

This experiment was a 2 (objecthood: *between* vs. *within*) × 2 (object type: eyeless, intact) within-subject design. In the intact condition, two rectangles were filled with Asian faces which were the same as in Experiment 1. In the eyeless condition, the same Asian faces were used but removed their eye region, as shown in Fig. 3 (A). Likewise, Experiment 2 also included the baseline condition and deployed only vertical rectangles. This experiment consisted of overall 192 trials (64 trials of the intact condition, 64 trials of the eyeless conditions, and 64 trials of the baseline condition).

The procedure was the same as Experiment 1. Experiment 2 lasted about 10 min.

### Results and analyses

The criterion for data exclusion was experimental duration longer than 20 min or accuracy lower than 85%. According to this criterion, no participant was excluded in Experiment 2.

Overall, incorrect responses (4.5%), RTs faster than 150 ms or slower than 1,000 ms (4.3%), and RTs outside 2 SDs of each condition (4.5%) were discarded. Thus, 86.7 % of the total trials were included in the statistical model.

Since we have illustrated how the horizontal bias overrides the OBA effect in Experiment 1 and the hypotheses were based on the corrected OBA analysis, the following part only reports the corrected OBA analyses. The traditional OBA analyses can be found in online material.

### Horizontal bias

The horizontal bias was calculated in the baseline condition. A one-way repeated-measures ANOVA was conducted on the RTs, with the orientation of attentional engagement (horizontal vs. vertical) as within-subject factors. The main effect was significant, *F*(1, 74) = 35.96, *p* < .001, *η*_p_^2^ = 0.327, with faster RTs in horizontal (593 ± 10 ms) than vertical engagement (614 ± 10 ms), showing a horizontal bias of 21 ms.

### Corrected OBA analyses

A 2 × 2 repeated-measures ANOVA was conducted on the RTs, with the objecthood (*corrected within* vs. *between*) and object type (intact vs. eyeless) as within-subject factors. The results are shown in Fig. 3 (B). The main effect of object type was not significant, *F*(1, 74) = 0.56, *p* = .456, *η*_p_^2^ = .008, indicating that there was no difference between the intact (593 ± 10 ms) and eyeless faces (591 ± 10 ms). The main effect of objecthood was significant, *F*(1, 74) = 11.00, *p* = .001, *η*_p_^2^ = .129, with shorter RTs in *between* (585 ± 9 ms) than *corrected within* (600 ± 11 ms), indicating the reversed OBA effect even after the correction. The interaction was not significant, *F*(1, 74) = 0.096, *p* = .759, *η*_p_^2^ = .001, suggesting that the corrected OBA effect was not affected by the presence of the eyes.

### Facial orientation

To further test whether face orientation has different influences on the OBA effect, a 2 × 2 × 2 repeated-measures ANOVA was conducted on RTs, with objecthood (*corrected within* vs. *between*), object type (intact vs. eyeless), and face orientation (upright vs. inverted) as within-subject factors. The results are shown in Fig. 3 (C and D). Like Experiment 1, the analysis showed that the main effect of the orientation and its interactions with the object type and objecthood were not significant, suggesting the orientation have little influence on the OBA.

#### ACC analyses

A 2 × 2 repeated-measures ANOVA was conducted on the ACC, with the objecthood (*within* vs. *between*) and object type (intact vs. eyeless) as within-subject factors. The main effect of the objecthood was significant, *F*(1, 74) = 7.53, *p* = .008, *η*_p_^2^ = .092, with a higher ACC in the *between* (95.7 ± 0.5%) than *within* (94.4 ± 0.6%), indicating the reversed OBA effect. The main effect of object type and objecthood were not significant.

In the baseline condition, a one-way repeated-measures ANOVA was conducted on the ACC, with the orientation of attentional engagement (horizontal vs. vertical) as within-subject factors. The main effect was significant, with a higher ACC in the horizontal engagement (97.1 ± 0.5%) than in vertical engagement (95.5 ± 0.5%), *F*(1,74) = 7.79, *p* = .007, *η*_p_^2^ = .092, indicating the presence of the horizontal bias. The corrected OBA was analyzed similarly to RT data. None of any main effect or interaction was found, suggesting no corrected OBA effect and its interaction with the object type.

In short, for the ACC data, the traditional OBA analysis showed the reversed OBA main effect but the corrected OBA analysis found neither the OBA main effect nor interaction.

## Discussion

This experiment presented intact and eyeless faces to explore the role of the eyes in the OBA effect. In the corrected OBA analysis, the reversed OBA effect was found in both eyeless and intact faces, with no interaction, suggesting that the eyes themselves do not modulate the OBA effect in faces. Likewise, the facial orientation showed no influence on the OBA effect. Combined with Experiment 1, both holistic and feature properties of faces do not seem to affect the object-based mechanism.

Overall, it is worth noting that this experiment found a reversed corrected OBA effect, rather than a null effect. Although this finding did not directly contradict our conclusion that faces do not affect attention in an object-based facilitation way, in General Discussion we provided possible explanations for this reversed OBA effect in conjunction with the mechanism of how faces affect attentional focus.

## Experiment 3

The results from Experiments 1 and 2 have left the question of why faces cannot guide attention in an object-based facilitation manner unanswered. To address this, we conducted Experiment 3 to further investigate this phenomenon. The purpose of this experiment was to examine the hypothesis that the null OBA effect could be attributed to the filtering cost (Kahneman et al., 1983) associated with attentional deployment within a face, which is perceptually and socially more relevant compared to mosaic patterns. Based on this assumption, we anticipated finding evidence of the OBA effect once the filtering cost associated with faces is reduced or eliminated.

To test this hypothesis, we modified the trial structure by shortening the screen permanence of faces to avoid any perceptual spatial or temporal overlapping with task-relevant targets. This ensured that no visible faces were presented on the screen when participants shifted their attention to the second target, hence eliminating (or at least dramatically reducing) any direct filtering cost. Noteworthy, previous findings showed that the OBA effect persists after a short period of object disappearance (Xie et al., 2021). Based on this evidence and in line with H3 we predict that the disappeared faces should elicit a significant OBA effect both in faces and in the mosaic objects.

### Method

#### Participants

As Experiment 3 was conducted in the laboratory, we reduced the sample size to 30. This sample size was determined by the Gpower for detecting a 2 × 2 within-subject design. Thirty participants (aged 19.5 ± 1.5 years, seven males, all right-handed) from Guangzhou University were recruited for Experiment 3, with a reward of 10 Yuan or course credit.

#### Apparatus

The experiment was conducted in a dim and sound-attenuated chamber. Participants were comfortably sitting about 60 cm from the LCD (resolution: 1,024 × 768 pixels, refresh rate: 100 Hz), with their eyes positioned at the same height as the center of the screen. The presentation of the stimulus and the manual response measurements were controlled with E-Prime 2.0 software (Psychological Software Tools, Inc., Pittsburgh, PA, USA).

#### Stimuli and materials

All the stimuli and materials were the same as in Experiments 1 and 2.

#### Design and procedure

**Fig. 4:**
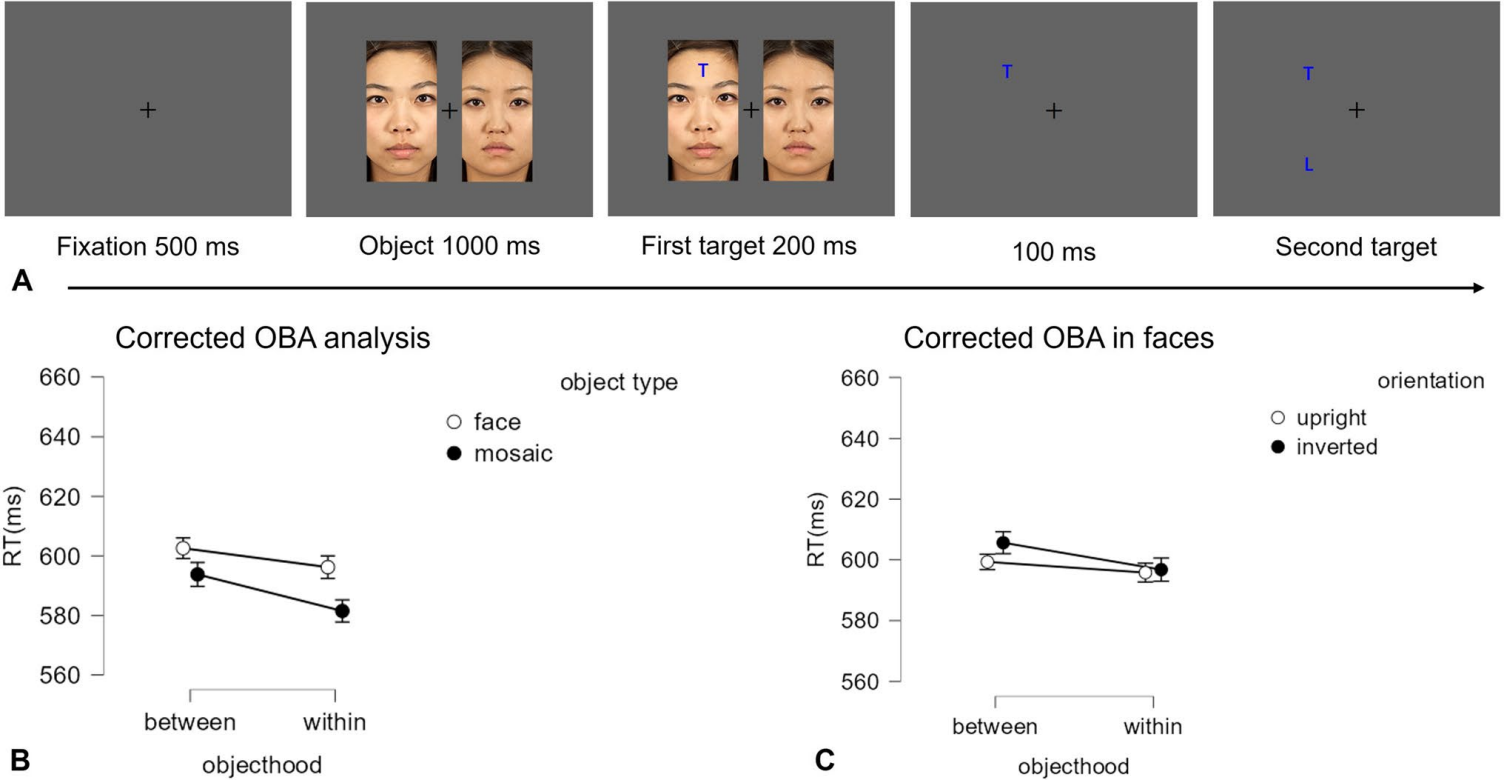
Panel **A** illustrates the procedure of each trial in Experiment 3. Panel **B** shows the corrected OBA results for faces and mosaic objects. Panel **C** shows the corrected OBA results in faces, with facial orientation as a factor

This experiment was a 2 (objecthood: *between* vs. *within*) × 2 (object type: mosaic, Asian face) within-subject design. Only vertical objects were presented, and the faces are presented both upright and inverted. The trial stricture was the same as Experiments 1 and 2 except that here the faces disappeared 200 ms after the onset of the first target. This remained on the screen for 100 ms before the onset of the second target. The sequence of each trial is shown in Fig. 4 (A).

This experiment consisted overall of 320 trials (64 baseline conditions, 64 face-*within*, 64 face-*between*, 64 mosaic-*within*, and 64 mosaic-*between*).

Before the formal experiment, participants performed a practice session in which they had to make eight consecutive correct responses. The whole experiment lasted about 20 min.

### Results and analyses

All participants were included in the analyses. Overall, incorrect response (4.7%), RTs faster than 150 ms or slower than 1,000 ms (3.3%), and RTs outside 2 SDs (5.2%) per condition were discarded. Thus, 86.8% of the total trials were included in the statistical model.

### Horizontal bias

The horizontal bias was calculated by the baseline condition. A one-way repeated-measures ANOVA was conducted on the RTs, with the orientation of attentional engagement (horizontal vs. vertical) as within-subject factors. The main effect was significant, *F*(1, 29) = 25.4, *p* < .001, *η*_p_^2^ = 0.467, with faster RTs in horizontal shifting (597 ± 11 ms) than vertical shifting (619 ± 12 ms), showing a horizontal bias of 22 ms. Given the significant horizontal bias, the correction was conducted.

### Corrected OBA analysis

A 2 × 2 repeated-measure ANOVA was conducted on the RTs, with the objecthood (*within* vs. *between*) and object type (mosaic, face) as within-subject factors. The results are shown in Fig. 4 (B). The main effect of object type was significant, *F*(1, 29) = 6.53, *p* = .016, *η*_p_^2^ = .184, with shorter RTs in the mosaic (588 ± 11 ms) than faces (599 ± 11 ms). The main effect of objecthood was significant, *F*(1, 29) = 6.53, *p* = .030, *η*_p_^2^ = .151, with shorter RTs in *within* (589 ± 12 ms) than *between* (598 ± 11 ms), suggesting the presence of the OBA effect. The interaction was not significant, *F*(1, 29) = 2.08, *p* = . 16, *η*_p_^2^ = .067.

### Facial orientation

A 2 × 2 repeated-measure ANOVA was conducted on the RTs, with the objecthood (*within* vs. *between*) and face orientation (upright vs. inverted) as within-subject factors. The results are shown in Fig. 4 (C). The main effect of objecthood was not significant, *F*(1, 29) = 2.06, *p* = .16, *η*_p_^2^ = .067, with similar RTs in the *within* (593 ± 13 ms) and *between* (602 ± 11 ms) condition. The main effect of face orientation was significant,* F*(1, 29) = 4.32, *p* = .047, *η*_p_^2^ = .130, with shorter RTs in the upright faces (598 ± 12 ms) than inverted faces (601 ± 11ms). The interaction was not significant,* F*(1, 29) = 0.64, *p* = .431, *η*_p_^2^ = .022, suggesting the orientation did not modulate the OBA effect.

#### ACC analyses

The mean accuracy was 95.3 %. The ACC analyses were conducted the same as Experiments 1 and 2. The results only showed a significant main effect of objecthood, *F*(1, 29) = 5.89, *p* = .022, *η*_p_^2^ = .169, with lower ACC (94.7 ± 0.8%) in *within* than *between* (95.6 ± 0.7%).

#### Analysis of the horizontal bias magnitude

To examine the stability of the magnitude of horizontal biases across the three experiments (32 ms in Experiment 1, 21 ms in Experiment 2, and 2 ms in Experiment 3), a one-way ANOVA with the Experiment as a between-subject factor was conducted. he results revealed no significant difference in magnitudes, *F*(1, 2) = 2.78, *p* = .065, *η*_*p*_^2^ = .031. Given the non-significant *p*-value, we further conducted a Bayesian ANOVA, which yielded a BF_10_ = 0.712, indicating that neither H_0_ nor H_1_ received support. That is, the relationship between the magnitudes of horizontal bias and the Experiment was inconclusive. Nevertheless, the existence of the horizontal bias indicated the necessity of the OBA correction.

## Discussion

The results of Experiment 3 were consistent with H3 that both faces and mosaic objects can elicit the OBA effect after their short disappearance. Note that the same faces were not able to elicit the OBA effect when presented in Experiments 1 and 2. By contrast, the results obtained in Experiment 3 were the opposite, supporting our hypothesis that the complex content of a face increases the filtering cost when attention operates within it. That is, when this filtering cost is eliminated by disappearing faces, the representation of faces successfully elicits the OBA effect.

## General discussion

This study aimed to address the question of why faces do not affect attention in an object-based manner. For this purpose, we put faces in the double-rectangle paradigm and used the OBA effect as an index to test how faces affect object-based components. Note that the OBA effect in this section was calculated after partialling out the horizontal bias (Xie et al., 2022). We refer to this derived measure (the corrected OBA effect) as the OBA effect in this section, for the sake of brevity.

Our results (Experiments 1 and 2) replicated the previous findings that faces elicit null or reversed OBA effects (Valenza et al., 2014; Xie et al., 2022) and further demonstrated that either race or the presence of the eyes was not critical for these results. Experiment 3 introduced a temporal lag between face offset and attentional shifting to avoid any potential attentional filtering cost due to the perceptual or social relevance of faces. As a result, Experiment 3 successfully revealed the presence of the OBA effect.

In summary, our study suggested that the null or reversed OBA effect in faces could not be explained by either local (eye region) or global (race and orientation) perceptual features themselves. Rather, our data suggest that the whole content of the face may operate by inducing a filtering cost, which slows the response and vanishes the OBA effect. Once this filtering cost is experimentally controlled for, faces can guide attention in an object-based manner.

### The correction for the OBA effect and the magnitude of horizontal bias

In this study, we introduce the baseline condition to calculate the horizontal bias of attention and incorporate the correction for the OBA effect. It should be noted that this is not the only method to deal with horizontal bias in the double-rectangle paradigm. In general, object orientations would be counterbalanced in this paradigm, which could counteract the advantage of attention shift in a certain orientation. Another approach is to use a detection task, which is less affected by horizontal bias (Chen & Cave, 2019). Although the detection task is widely adopted in OBA studies, it inevitably introduces valid trials, which are not directly related to the analyses of the OBA effect. In our study, the online data collection requires a more trial-saving task and thus we sought the two-target comparison task. Together with the specificity of faces not being suitable for horizontal orientation, we presented faces only vertically and measured the horizontal bias of attention for the correction. Given these reasons, we chose this particular method for our data analysis.

Another relevant aspect is the magnitude of the horizontal bias, which appears to play a role in determining whether we observe a reversed, null, or standard OBA effect. While the presence of the horizontal bias was consistently observed across experiments, the analysis comparing its magnitude between experiments yielded inconclusive results.

However, it is important to clarify that the inconclusive relationship between the magnitude of the horizontal bias and the Experiment does not imply the ineffectiveness of our correction method. The rationale behind the correction is that the horizontal bias would impact the OBA effect and the “pure” OBA effect can be obtained when this bias is excluded. Based on our results that the vertical mosaic objects elicited null or reversed OBA effect, we predicted that this result was due to the horizontal bias, which was shown in the baseline condition. With the implementation of the correction, we predict that the OBA effect would return to a more "normal" level, thereby demonstrating that the reversed OBA effect is indeed attributable to the horizontal bias. Otherwise, if the corrected OBA effect still is reversed or null, it will indicate that (1) the correction is ineffective or (2) inherent characteristics of the materials elicit a null or reversed OBA effect. Considering the presence of a null OBA effect and a significant corrected OBA effect in mosaic objects, we concluded that the correction method we employed is effective. Therefore, the absence of a reversed corrected OBA effect in faces is more likely attributed to the properties of faces themselves, rather than a methodological limitation of the correction. Furthermore, it is important to emphasize that the measurement of the horizontal bias and the correction for the OBA effect were conducted at the individual level. In other words, the individual differences in horizontal bias only influence the corrected OBA effect for specific participants. Thus, the magnitude of the horizontal bias does not directly determine the OBA effect, but rather acts as an intermediate variable that varies among individuals.

In summary, although the stability of the magnitude of the horizontal biases across experiments remains inconclusive, our findings indicate that the bias itself does not directly influence the underlying mechanism of the OBA.

### The effect of race

Numerous studies have been carried out on the impact of race on face processing. The fact that same-race faces are better to be recognized than other-race faces is referred to as the *other-race effect* (see Young et al., 2012, for a review). However, the effect of race on face processing could also be seen as an other-race advantage, which has been found when searching for an other-race face among same-race faces (Levin, 1996, 2000; Sun et al., 2013). Although Experiment 1 did not show an interaction between race and the OBA effect, Asian participants showed faster overall RTs in Caucasian than Asian faces, indicating the presence of other-race advantage.

In short, given that no object-based facilitation was observed in both Asian and Caucasian faces, we concluded that race does not determine the OBA patterns in faces.

### The effect of the eyes

It is widely recognized that the eyes play a crucial role in face processing and serve as a critical area from which a wealth of information can be extracted (Itier & Batty, 2009). When the eye region was occluded or removed, the ability of face detection, perception, classification, and recognition was dropped (Caldara et al., 2005; Itier et al., 2007; Lewis & Edmonds, 2003). However, when the eye region of the faces was blurred in Experiment 2, we found that attention shifting occurred in a similar way to that with the intact face. Surprisingly, neither the overall RTs nor the reversed OBA effect were affected by the presence or absence of eyes, suggesting that the feature property of faces like the eye does not modulate the object-based mechanism.

While this result may seem unexpected based on previous literature, it was in line with evidence that faces with or without eyes can elicit comparable activation of the Fusiform Face Area (FFA) (Tong et al., 2000). This finding implied that other facial regions besides the eyes are also crucial for face processing, which could potentially account for our results.

Notably, our conclusion was opposed to the results of Yan et al. (2022), who reported the OBA effect when the eyes with direct eye gaze overlapped on cups (Yan et al., 2022). However, it is worth noting that in this study, the cup itself was also an object that was expected to elicit the OBA effect. For this reason, this study cannot distinguish whether the OBA effect is caused by the cup or the eyes themselves. In addition, no “eyeless” condition was included in the experimental design. As a result, determining whether the presence of eyes is a fundamental feature for eliciting the OBA effect cannot be easily addressed by their study alone.

### The effect of facial orientation

Another well-known effect in face processing is the face inversion effect, which refers to the impaired recognition of inverted compared with upright faces (Valentine, 1988). However, our results again contradicted this established knowledge. In this study, facial orientation showed neither main effect nor interaction with other factors, suggesting that the holistic properties of faces can not affect the object-based mechanism.

It is noteworthy that our experimental task was not conceived to directly assess the presence of the inversion effect. Indeed, while this effect has been traditionally investigated by simple stimulus detection tasks. However, we used the two-target comparison task, making it difficult to directly compare our findings with the previous studies. In addition, several studies suggested that inverted faces are not processed differently from upright faces (Murphy et al., 2020; Sekuleret al., 2004; Willenbockel et al., 2010). For these reasons, it is not surprising that the inverted and upright faces may have similar effects on the object-based component.

### The influence of task-irrelevant faces

Overall, our results seemed to contradict the extensive evidence that supported well-known experimental phenomena related to face processing (Itier & Batty, 2009; Valentine, 1988; Young et al., 2012), but it should be borne in mind that our experiments present several methodological differences compared to extant literature. Besides the aforementioned differences, such as the type of task employed, another important distinction in our experiments was that the faces were task-irrelevant. This implies that any effects on attentional shifting may have occurred implicitly. While we did not directly address this issue, our findings suggested that the effects of race, eye region, and face orientation may not be as critical in task-irrelevant contexts as they are when faces are task-relevant.

Although some studies suggested that task-irrelevant faces capture attention (Bindemann et al., 2007; Sato & Kawahara, 2015), Pereira and colleagues showed that when factors such as stimulus, task, and oculomotor were controlled, attention is no longer biased by task-irrelevant faces compared with non-face objects in the dot-probe task (Pereira et al., 2019, 2020). Similarly, task-irrelevant faces and non-face objects showed equal interference effects on the Stroop task, suggesting the task-irrelevant faces did not attract more attention (Henschel et al., 2021). Based on these findings, it is plausible to propose that the task-irrelevant faces in our experiments may have significantly reduced the influences of race, eye region, and facial orientation on face processing, thus explaining the absence of the traditionally reported effects.

#### The OBA effect in faces

In the double-rectangle paradigm, several studies found a significant OBA effect in faces (Hu et al., 2021; Song et al., 2021; Yan et al., 2022). These studies posited that faces, being real-world objects with inherent object properties, naturally elicit OBA effects.

However, some studies have found no typical OBA effect in faces, which must be attributed to either shortened RTs in the *between* condition, the prolonged RTs in the *within* condition, or both. Valenza et al. (2014) argued that the null OBA effect for faces may be due to the shortened RTs when moving attention between faces (Valenza & Calignano, 2021; Valenza et al., 2014). They proposed that the display of two faces in the visual field induces a larger attentional focus, leading to faster attentional shifts between faces. Opposite to this view, a previous study (Xie et al., 2022) found prolonged RTs associated with the *within* condition. Here we replicate our previous findings. Anyway, although advocating different explanatory hypotheses, neither our study nor the study by Valenza and colleagues reported evidence of OBA for faces. In any case, one should consider that the different measures used between our study (RTs) and the Valenza et al. study (saccadic latencies) make difficult any reliable methodological comparison, preventing a thorough examination of the mechanisms accounting for the null OBA effect.

Those studies that obtained a significant OBA effect in faces went beyond our research question and addressed the hypothesis that the OBA effect may be modulated by specific, local facial features such as emotion or eye gaze (Hu et al., 2021; Song et al., 2021; Yan et al., 2022). Although these studies obtained the OBA effect in faces, they neither included non-face objects for comparison nor considered horizontal bias. In addition, these studies seemed to repeatedly present the same face throughout the experiment, which would lead to undesired influences such as lower ecological validity. In contrast, our study addressed these limitations by including non-face objects as a control condition and aiming to maintain the ecological validity of faces. Specifically, our results were obtained by considering the horizontal bias and increasing the stimulus variability (less repetition of the same faces in the whole experiment). Therefore, these differences in experimental design may account for the divergent OBA patterns observed in these previous studies compared to ours.

#### The filtering cost affects the OBA effect in faces

The filtering cost (Kahneman et al., 1983), a phenomenon first introduced in visual search research, refers to the delay in attentional allocation caused by salient distractors that compete for attentional resources (Becker, 2007; Folk & Remington, 1998; Folk et al., 2009). The content of faces is likely to induce filtering costs due to their perceptual complexity and social relevance. Hence, the filtering can induce prolonged RTs when shifting attention within a face. Kahneman et al. (1983) suggested that filtering costs can be reduced or eliminated by preventing potential distractors from diverting attention away from the target. In Experiment 2, it was found that faces without eyes still failed to elicit the OBA effect, indicating that the presence of eyes alone was not sufficient to induce the filtering cost. This suggests that local features, such as the eyes, may not play a critical role in generating the filtering cost. Consequently, the global pattern or configuration of faces may be more influential in inducing the filtering cost. To explore this possibility, Experiment 3 involved making the entire face disappear, eliminating any visible elements that could capture attention during attention shifting. In this case, the OBA effect was observed. This finding supports the notion that the filtering cost associated with faces is the underlying cause of the null or reversed OBA effect previously observed.

However, although the results of Experiment 3 showed both mosaic objects and faces elicited significant OBA effects with equal magnitude, our findings also revealed that shifting attention within a face is slower than within a mosaic object. That is, the disappearance of faces can not eliminate but only reduce the filtering cost. In addition, given that the OBA effect has been reported in objects that also share complex content such as banknotes (Zhao et al., 2020), the filtering cost account may only be specific to faces because of its unique social relevance.

In addition, what the filtering cost account suggested is that the entire face contents led to the absence of the OBA effect. The removal of the faces may be driving the difference in performance for faces compared to mosaic objects. That is, some other factors that may cause the absence of the OBA effect in faces may be gone along with the removal of faces. For example, attentional engagement and disengagement with a face may have a different impact compared to a mosaic object, which could be a potential factor influencing the OBA effect, as proposed by the attentional shifting hypothesis (Brown & Denney, 2007). Hence, the filtering cost account may not provide a specific explanation for the absence of the OBA effect in faces but rather offers a broader perspective on why it is absent. The specific mechanisms underlying the absence of the OBA effect in faces require empirical research.

In summary, we proposed that faces do not influence attention in an object-based facilitation manner because of the filtering cost of the whole face content.

#### Absence or reversal of OBA effect in faces?

In Experiment 1, no significant OBA effect was observed in faces, but the reversed OBA effect was observed in Experiment 2. These different OBA patterns in faces may be attributed to the differences in design between Experiments 1 and 2. In Experiment 1, Asian faces, Caucasian faces, and mosaic objects were presented randomly, while Experiment 2 presented only Asian faces in every trial. According to the attentional focus hypothesis proposed by Valenza et al. (2014), human faces could induce a larger focus than non-face objects. Therefore, the presentation of faces trial by trial (Experimental 1) may induce a larger attentional focus compared with the presentation of faces mixed with non-face objects inter-block (Experiment 2). The larger attentional focus would reduce the cost of shifting attention between faces. Consequently, the faster RTs in *between* may have led to the reversed OBA effect in Experiment 2.

Taken together, the results of Experiments 1 and 2 did not contradict our conclusion that faces cannot exert object-based facilitation in attention.

## Conclusion

In conclusion, this study provides evidence that faces do not exert object-based facilitation in the double-rectangle paradigm, irrespective of race or the presence of eyes. The absence of the object-based facilitation effect in faces may be attributed to the filtering cost associated with the global properties of faces, which slows the response when shifting attention within a face and leads to the null or reversed OBA effect.
